# Characterization of the complete mitochondrial genome of *Cynoglossus nanhaiensis* (Pleuronectiformes: Cynoglossidae)

**DOI:** 10.1080/23802359.2020.1781582

**Published:** 2020-06-26

**Authors:** SiLin Tian, Chen Wang, Yunrong Yan, Xiao Chen

**Affiliations:** a College of Marine Science, South China Agriculture University, Guangzhou, China; b Guangdong Laboratory for Lingnan Modern Agriculture, South China Agriculture University, Guangzhou, China; c College of Fisheries, Guangdong Ocean University, Zhanjiang, China

**Keywords:** Mitochondrial genome, *Cynoglossus nanhaiensis*, phylogenetic position

## Abstract

In this study, the complete mitochondrial genome of *Cynoglossus nanhaiensis* was determined. The total length of the mitochondrial genome is 17,130 bp, including 13 protein-coding genes, 22 tRNA genes, two rRNA genes, and two noncoding regions. The gene rearrangement of tRNA^Gln^ gene and control region (CR) were detected, forming a unique gene order of CR-Ile-Gln-Met. Maximum-likelihood and Bayesian inference method are used to perform the phylogenetic analysis, and the result reveals a close relationship between *C. nanhaiensis* and *Cynoglossus itinus*.

*Cynoglossus nanhaiensis* widely distributed in coastal waters of the South China Sea and Viet Nam, belongs to the family Cynoglossidae of Pleuronectiforms. It features the combination of three ocular-side lateral lines, eight caudal-fin rays, a single (blind side) pelvic fin, and two ocular-side nostrils. This species was first described in 2016 (Wang et al. 2016). Currently, there are still rare research on *C. nanhaiensis* around the world. This study analyzes the mitochondrial genome characters and phylogenetic position of *C. nanhaiensis* for the first time, aiming to provide molecular data for its accurate taxonomy and evolutionary history.

The specimen of *C. nanhaiensis* (Voucher No. BH201003146) was collected from Beihai, China and stored in College of Marine Sciences, South China Agricultural University. Primers were designed to amplify the entire mitogenome, and the data analysis method was according to the previous study (Yu et al. 2019). Besides *C. nanhaiensis*, we chose *Scophthalmus maximus* as the outgroup and 17 other species of the family Cynoglossidae to construct the phylogenetic tree. All mitogenome data were downloaded from Genbank. The concatenation of 12 protein-coding genes totally 11,022 bp, except for *ND6*, was used to perform the analysis. Maximum-likelihood estimation and Bayesian inference approaches were used to infer phylogenetic trees by IQ-TREE 1.6.12 (Nguyen et al. 2015) and MrBayes 3.2.6 (Ronquist et al. 2012) software.

The complete mitochondrial genome of *C. nanhaiensis* was 17,130 bp in length (Genbank accession: MT117229), containing 13 protein-coding genes, 22 tRNA genes, two rRNA genes, one control region (CR), and origin of light-strand replication(OL). The overall base composition was 30.4% A, 29.6% T, 15.1% G, and 24.8% C, exhibiting a strong A + T bias (60.0%). The length of 13 protein-coding genes ranged from 165 bp (*ATP8*) to 1860 bp (*ND5*). Most of the protein-coding genes were initiated by ATG codon while *COI* and *ND3* genes began with GTG and ATT, respectively. Moreover, most of the genes were terminated by TAA codon except *ND2* using TAG as a stop codon. All 22 tRNA genes, with length ranging from 66 bp to 74 bp, were identified to be folded into typical secondary structures using tRNAscan-SE 2.0 (Lowe and Chan 2016). The 12S rRNA (949 bp) and 16S rRNA (1698 bp) were located between tRNA^Phe^ and tRNA^Leu^ (UUR) and were separated by tRNA^Val^. The origin of light-strand (37 bp) was located between tRNA^Asn^ and tRNA^Cys^ and predicted to form a stable 13-bp stem and 12-bp loop. Especially, instead of its typical location (between tRNA^Pro^ and tRNA^Phe^), the control region translocated into the position between *ND1* and tRNA^Gln^ genes, leaving a 24 bp trace fragment in the original place. Additionally, the tRNA^Gln^ gene not only translocated into the position between CR and tRNA^Ile^ instead of between tRNA^Ile^ and tRNA^Met^ but also inverted to the H-strand from the L-strand, which could result in downstream effects. In general, we found a unique gene order of CR-Ile-Gln-Met, which was different from the typical gene order of CR-Gln-Ile-Met in other species. The results suggested the CR, tRNA^Gln^ and tRNA^Ile^ loci might be the frequent sites of rearrangement.

The fact that the Bayesian and Maximum-Likelihood analysis produce an identical topology with similar branch lengths, strong bootstraps and posterior probabilities values suggests that the phylogenetic tree was well-supported (Figure 1). It is shown that *C. nanhaiensis* cluster with *C. itinus* to form the basal clade in a monophyletic group including three species of *Paraplagusia* and nine *Cynoglossus* species. Then the *Paraplagusia* + *Cynoglossus* clade clusters with a clade including the other four *Cynoglossus* species. This result corresponds to the previous results (Ren et al. 2016; Wei et al. 2016; Song et al. 2019) that *Paraplagusia* was a new clade which occurred later than *Cynoglossus*. Moreover, the *Paraplagusia* clade has a close phylogenetic relationship with *C. puncticeps*. In traditional taxonomy, The *Paraplagusia* and *Cynoglossus* were firstly distinguished by the possession of a series of fringes on the lips on the ocular side, and two genera were very similar in all other features (Menon 1977). The skeletal characteristics were also used to correct the original classification basis (Chapleau et al. 1991). These morphological taxonomy evidences also support the molecular phylogenetic relationship between *Paraplagusia* and *Cynoglossus* in this study. Furthermore, the results in this study confirm that the entire *Symphurus* genus shows the most distant relationship with other species in Cynoglossidae and is separated at the earliest stage. It is suggested that the mitogenome data could elucidate the clearer phylogenetic classification of Cynoglossinae species.

**Figure 1.**
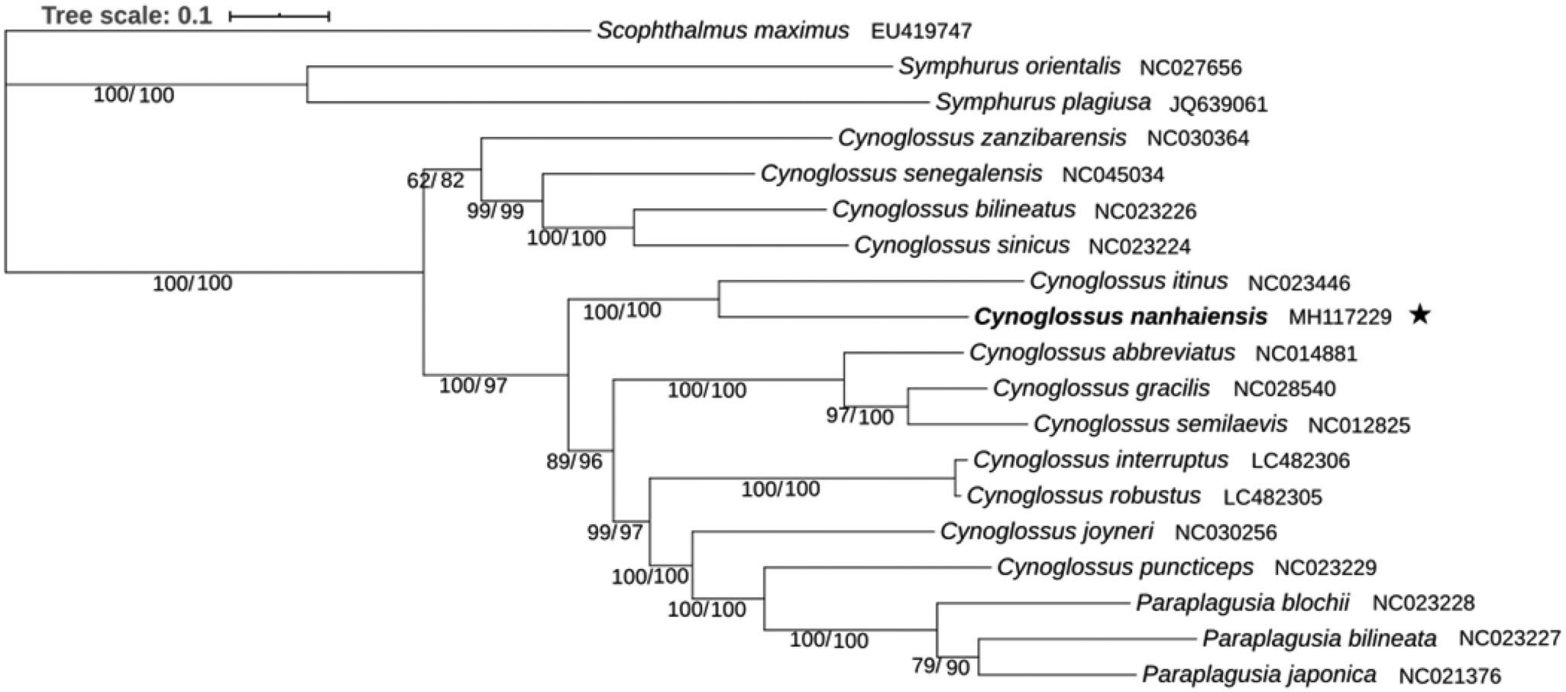
Phylogenetic position of *Cynoglossus nanhaiensis* based on a comparison with the mitochondrial genome of 18 species. Bootstrap support (left) and Bayesian posterior probability (right) values are displayed next to the nodes.

## Data Availability

The data that support the findings of this study are openly available in GenBank at https://www.ncbi.nlm.nih.gov, reference number MT117229.

## References

[CIT0001] Chapleau F, Renaud CB, Kailola PJ. 1991. *Paraplagusia longirostris*, a new flatfish (Cynoglossidae) from Australia and Papua New Guinea. Jap Jour Ich. 38(3):239–244.

[CIT0002] Lowe TM, Chan PP. 2016. tRNAscan-SE On-line: integrating search and context for analysis of transfer RNA genes. Nucleic Acids Res. 44(W1):W54–W57. 27174935 10.1093/nar/gkw413 PMC4987944

[CIT0003] Menon AGK. 1977. A systematic monograph of the tongue soles of the genus *Cynoglossus* Hamilton-Buchanan (Pisces: Cynoglossidae). Smithson Contrib Zool. 238(238):1–129.

[CIT0004] Nguyen L-T, Schmidt HA, von Haeseler A, Minh BQ. 2015. IQ-TREE: a fast and effective stochastic algorithm for estimating maximum-likelihood phylogenies. Mol Biol Evol. 32(1):268–274. 25371430 10.1093/molbev/msu300 PMC4271533

[CIT0005] Ren LH, Xu T, Sun GH. 2016. The complete mitochondrial genome of *Cynoglossus joyneri* (Teleostei: Pleuronectiformes). DNA B. 2(2):581–582. 10.1080/23802359.2016.1192512 PMC7799481 33473651

[CIT0006] Ronquist F, Teslenko M, van der Mark P, Ayres DL, Darling A, Höhna S, Larget B, Liu L, Suchard MA, Huelsenbeck JP. 2012. MrBayes 3.2: efficient Bayesian phylogenetic inference and model choice across a large model space. Syst Biol. 61(3):539–542. 22357727 10.1093/sysbio/sys029 PMC3329765

[CIT0007] Song HY, Kim J-K, Jo S, Jung S-H, Kim B, Choi YJ, Yoo JS, Lee D-S. 2019. Gene rearrangements in the mitochondrial genome of robust tonguefish, *Cynoglossus robustus* (Pleuronectiformes: Cynoglossidae) and a comparative analysis with other *Cynoglossus* fishes. Mitochondrial DNA B. 4(2):2924–2925. 10.1080/23802359.2019.1637297 PMC7720723 33366553

[CIT0008] Wang ZM, Thomas AM, Kong XY. 2016. A new species of tongue sole (Pisces: Pleuronectiformes: Cynoglossidae: *Cynoglossus*) from coastal waters of the South China Sea. Int J Biol Macromol. 129(1):129–143.

[CIT0009] Wei M, Liu Y, Guo H, Zhao F, Chen S. 2016. Characterization of the complete mitochondrial genome of *Cynoglossus gracilis* and a comparative analysis with other Cynoglossinae fishes. Gene. 591(2):369–375. 27312953 10.1016/j.gene.2016.06.023

[CIT0010] Yu Y, Zhang H, Yang C-M, Chen X, Peng X, Qin S. 2019. Complete mitochondrial genome and the phylogenetic position of the thornback cowfish (*Lactoria fornasini*). Mitochondrial DNA B. 4(1):1951–1952.

